# Supplementary material for: New Cobalt (II) Complexes with Imidazole Derivatives: Antimicrobial Efficiency against Planktonic and Adherent Microbes and In Vitro Cytotoxicity Features
Source: Molecules. 2020 Dec 24;26(1):55. doi: 10.3390/molecules26010055 (PMC7796409; doi:10.3390/molecules26010055)
Supplement: Supplementary file 1 [file molecules-26-00055-s001.pdf]

molecules

Supplementary materials

New cobalt (II) Complexes with Imidazole Derivatives Developed as Effective Antimicrobial and Antitumor Species

Alina Fudulu, Rodica Olar, Cătălin Maxim, Gina Vasile Scăeţeanu, Coralia Bleotu, Lilia Matei, Mariana Carmen Chifiriuc and Mihaela Badea

Table S1. Selected angles (°) in (**1**)-(**3**).

(1)				(2)				(3)			
O(3)	Co(1)	O(1)	106.14(11)	O(5)	Co(1)	O(1)	106.23(14)	N(3)	Co(1)	C(1A)	97.19(12)
N(3)	Co(1)	O(1)	92.87(12)	N(2)	Co(1)	O(1)	115.97(15)	N(2)	Co(1)	C(1A)	133.93(14)
N(3)	Co(1)	O(3)	113.21(12)	N(2)	Co(1)	O(5)	103.02(14)	N(2)	Co(1)	N(3)	99.81(13)
N(1)	Co(1)	O(1)	111.55(13)	N(1)	Co(1)	O(1)	114.02(16)	O(1)	Co(1)	C(1A)	114.13(12)
N(1)	Co(1)	O(3)	120.51(12)	N(1)	Co(1)	O(5)	100.37(15)	O(1)	Co(1)	N(3)	103.37(12)
N(1)	Co(1)	N(3)	109.05(13)	N(1)	Co(1)	N(2)	114.77(17)	O(1)	Co(1)	N(2)	103.02(13)
								O(3)	Co(1)	C(1A)	29.67(11)
								O(3)	Co(1)	N(3)	102.88(12)
								O(3)	Co(1)	N(2)	104.44(13)
								O(3)	Co(1)	O(1)	137.73(11)
								O(4)	Co(1)	C(1A)	29.19(11)
								O(4)	Co(1)	N(3)	89.35(11)
								O(4)	Co(1)	N(2)	162.65(12)
								O(4)	Co(1)	O(1)	88.97(10)
								O(4)	Co(1)	O(3)	58.86(10)
								O(2)	Co(1)	C(1A)	85.25(11)
								O(2)	Co(1)	N(3)	160.44(12)
								O(2)	Co(1)	N(2)	92.16(12)
								O(2)	Co(1)	O(1)	58.53(10)
								O(2)	Co(1)	O(3)	88.86(10)
								O(2)	Co(1)	O(4)	83.37(10)

N(11)	Co(3)	N(10)	105.42(14)
O(12)	Co(3)	N(10)	103.76(14)
O(12)	Co(3)	N(11)	102.41(13)
O(9)	Co(3)	N(10)	97.40(13)
O(9)	Co(3)	N(11)	104.84(14)
O(9)	Co(3)	O(12)	139.35(15)
N(7)	Co(2)	N(6)	100.31(14)
O(5)	Co(2)	N(6)	102.57(13)
O(5)	Co(2)	N(7)	110.79(14)
O(7)	Co(2)	N(6)	110.42(13)
O(7)	Co(2)	N(7)	100.76(13)
O(7)	Co(2)	O(5)	128.74(13)

Table S2. Continuous Shape Measures for the coordination polyhedron around the Co(II) atom.

Geometry	Co1 (compound 1)	Co1 (compound 2)	Co3 (compound 3)
PP-5	30.990	34.297	30.063
vOC-5	7.796	8.032	4.109
TBPY-5	3.318	3.020	5.782
SPY-5	5.643	5.918	3.225
JTBPY-5	4.249	3.820	6.493

Geometry	Co1 (compound 3)
HP-6	29.330
PPY-6	23.830
OC-6	4.345
TPR-6	12.152
JPPY-6	26.949

Geometry	Co2 (compound 3)
SP-4	30.034
T-4	0.905
SS-4	5.744
vTBPY-4	2.774

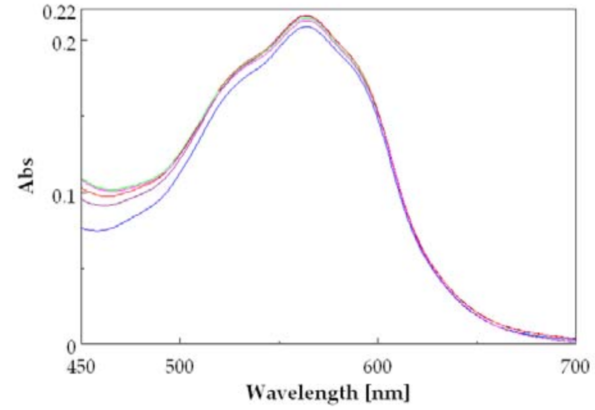

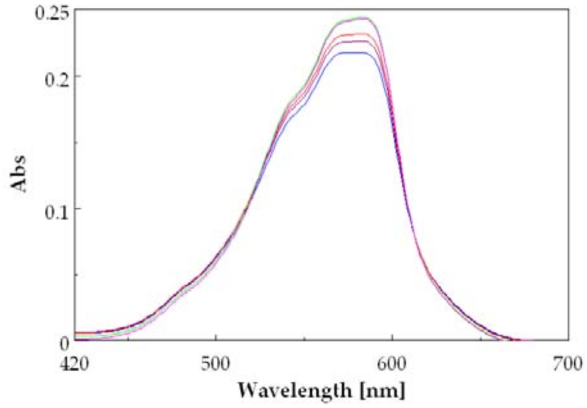

(**1**) (**2**)

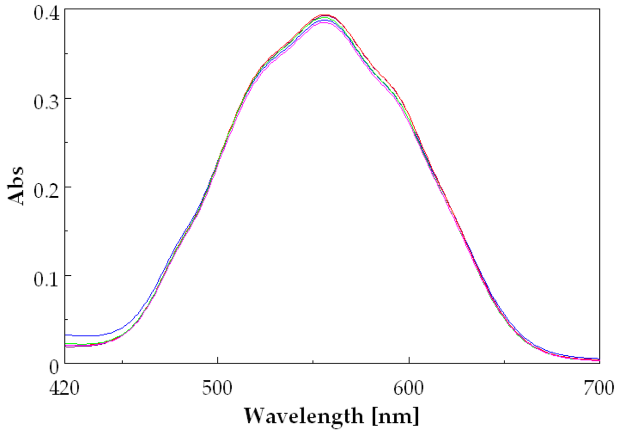

(**3**)

Figure S1. UV-Vis spectra in DMSO solution of complexes (**1**)-(**3**): (blue – 0 min, purple – 6 h, red – 12 h, green – 24 h, magenta -48h).

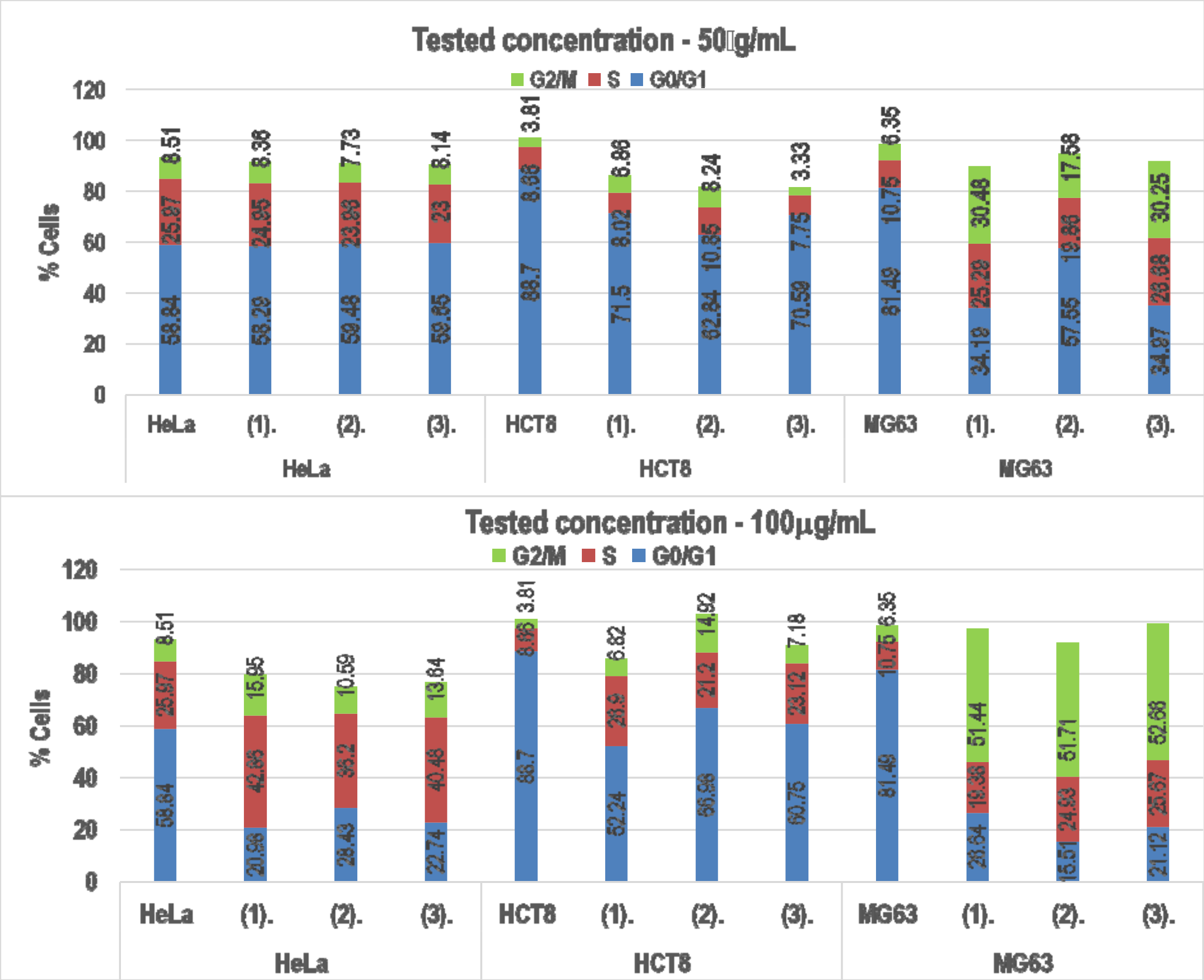

Figure S2. Effect of complexes (**1**) - (**3**) on cell cycle phases in HeLa, HCT8 and MG63 cells. The results are presented as percent of cells in different phases of the cell cycle.
